# Plucked human hair as a tissue in which to assess pharmacodynamic end points during drug development studies

**DOI:** 10.1038/sj.bjc.6602558

**Published:** 2005-05-10

**Authors:** D R Camidge, K R Randall, J R Foster, C J Sadler, J A Wright, A R Soames, P J Laud, P D Smith, A M Hughes

**Affiliations:** 1 Edinburgh Cancer Centre, Western General Hospital, Edinburgh EH4 2XU, UK; 2 AstraZeneca, Alderley Park, Macclesfield, Cheshire SK10 4TG, UK; 3 Syngenta, CTL, Alderley Park, Macclesfield, Cheshire SK10 4TJ, UK

**Keywords:** hair, drug development, pharmacodynamic

## Abstract

We have demonstrated the feasibility of detecting and quantifying six cell-cycle-related nuclear markers (Ki67, pRb, p27, phospho-p27 (phosphorylated p27), phospho-pRb (phosphorylated pRb), phospho-HH3 (phosphorylated histone H3)) in plucked human scalp and eyebrow hair. Estimates of the proportion of plucked hairs that are lost or damaged during processing plus the intra- and intersubject variability of each nuclear marker with these techniques are provided to inform sizing decisions for intervention studies with drugs potentially impacting on these markers in the future.

Plucked human hairs contain proliferating cells within the hair sheath (Moll, 1996; Gho *et al*, 2004); they are therefore potentially attractive as an easily accessible tissue in which to assess the pharmacodynamic (PD) effects of drugs that interfere with cell proliferation. Skin biopsies have already been used to similar ends during the development of a number of agents targeted against the epidermal growth factor receptor (Albanell *et al*, 2002; Malik *et al*, 2003; Salazar *et al*, 2004; Vanhoefer *et al*, 2004). Plucked hairs would offer certain advantages over skin biopsies for these purposes including improved tolerability, a greater potential for multiple time-point analyses and the possibility of a higher signal within hair follicles for certain key biomarkers compared to the cells of the epidermis (Albanell *et al*, 2002). We have developed a technique for quantifying cell-cycle-related proteins within the sheath cells of plucked human hair.

## MATERIALS AND METHODS

### Trial design

In all, 12 healthy Caucasian males, within the age range 18–45 years, and with scalp hair greater than or equal to 5 mm in length, were recruited. Following thorough combing to remove any loose hairs, five suitable hairs with visible bulbs were plucked from each of the left eyebrow, right eyebrow and four different scalp sites using a pair of blunt-nosed forceps. Each set of five hairs was trimmed with scissors to a workable length (approximately 1 cm), retaining the bulb end of each hair, and then immersed in prelabelled 1.5 ml microcentrifuge tubes containing 1 ml 100% acetone at 4°C. Plucked hairs without visible bulbs were discarded and not processed.

### Immunohistochemistry (IHC) and signal quantification

#### Hair processing

Following acetone fixation for 10 min, each hair was air-dried for 10 min and then placed, in batches of five from each sampling site, in 1 ml of 0.5 M, pH 7.6, Tris-buffered saline.

#### Hair IHC

In our hands, it was not possible to paraffin embed and then section the hairs for later staining without producing significant loss or damage of the fragile material; we therefore developed techniques based on staining intact hairs followed by rigid epoxy resin embedding and then sectioning.

Briefly, a peroxidase block (5 min at room temperature), followed by a nonspecific protein block (30 min at room temperature), was employed on the intact hairs. A primary antibody, or buffer for negative controls, was then applied (anti-Ki67 at 1 : 100 for 1 h at room temperature, *DAKO M7240*; anti-pRb at 1 : 200 for 48 h at 4°C, *DAKO 7131*; anti-phospho-pRb (phosphorylated pRb) at 1 : 200 for 48 h at 4°C, *BIOSOURCE 44-584*; anti-p27 at 1 : 100 for 48 h at 4°C, *Research Diagnostics RDI-P27CABR*; anti-phospho-P27 (phosphorylated p27) at 1 : 100 for 48 h at 4°C, *UPSTATE 06-996* and phospho-HH3 (phosphorylated histone H3) at 1 : 200 for 48 h at 4°C, *Upstate 06-570*). The specificity of all antibodies was validated independently by the detection of single bands on Western blots of MCF7 cell extracts, by signal abolition on blocking peptide preincubation (where available) and by signal abolition on alkaline phosphatase preincubation for phospho-specific epitopes. The secondary system used was the *EnVision*™ *Plus* HRP (horse radish peroxidase) system (mouse and rabbit). The *EnVision*™ polymer was incubated with the hairs for 30 min at room temperature. The chromogen DAB (3′-diaminobenzidine) was applied for 5 min at room temperature. The hairs were then individually mounted in an aqueous mount, and a coverslip applied. All washes were with 0.5 M, pH 7.6, Tris-buffered saline with 0.5% Tween-20.

#### Hair staging

**Figure 1:**
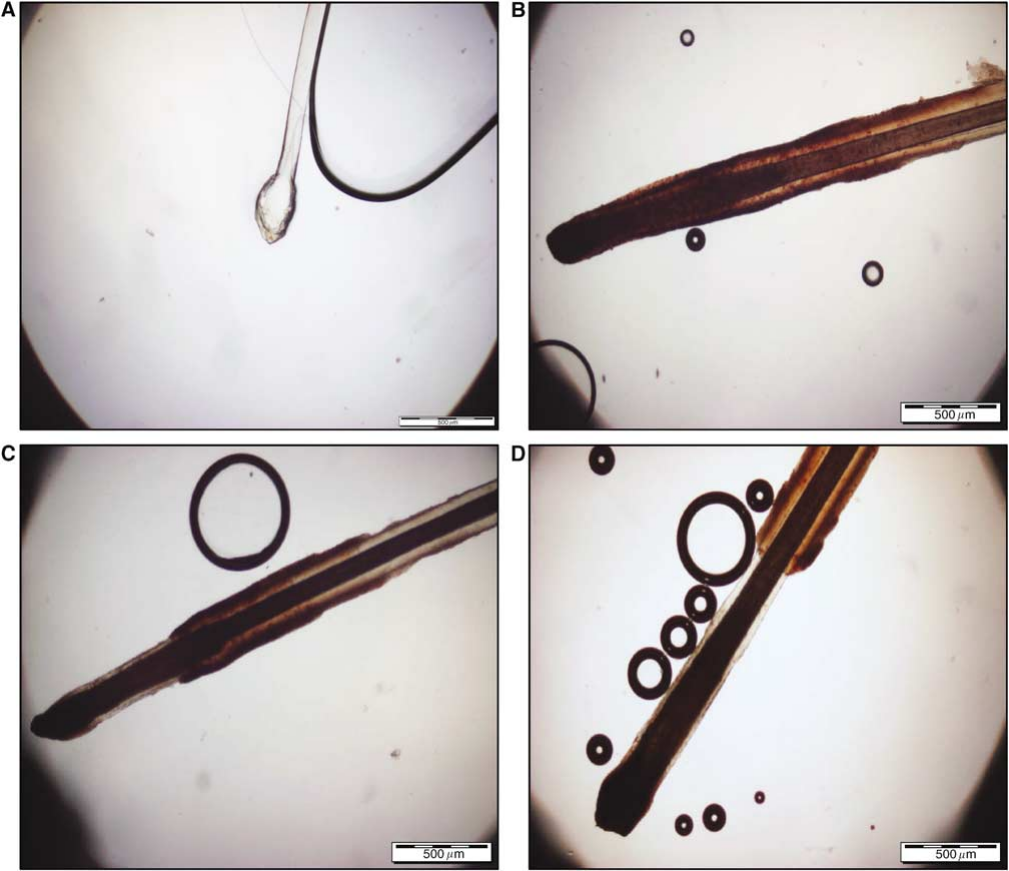
Exploratory staging system used within study on plucked scalp hairs (Ki67-stained hairs). Stage 0 (**A**)=sheath encompassing bulb. Stage 1 (**B**)=sheath <150 *μ*m from the base of the bulb. Stage 2 (**C**)=sheath 150–699 *μ*m from the base of the bulb. Stage 3 (**D**)=sheath >700 *μ*m from the base of the bulb. The scale bars represent 500 *μ*m.

The hairs were examined by light microscopy to evaluate nuclear staining in terms of its presence/absence, site of staining and stage of hair. Each hair with a visible bulb and root sheath was photographed and staged (0, 1, 2 or 3), according to a bespoke system based on the distance of the lower margin of the sheath from the base of the bulb (Figure 1). Stage 0=sheath encompassing the bulb, stage 1⩽150 *μ*m, stage 2=150–699 *μ*m and stage 3⩾700 *μ*m. Hairs noted to be without visible bulbs and sheaths that had not been excluded previously at the initial by-eye examination were discarded at this point. Every hair with both a bulb and sheath present was taken forward for embedding into epoxy resin.

#### Hair embedding

The hairs on the slides were placed in individual dishes of distilled water overnight to float off the coverslips. The hairs were loaded onto an automated EMTP processor (*Leica*) for dehydration and impregnation with freshly prepared resin (*Taab Araldite Cy212 Resin*) at 40°C for a minimum of 1 h. The resin was then polymerised at 65°C overnight.

#### Hair sectioning

The whole of each hair was sectioned tangentially at 2 *μ*m (approximately 120 sections per hair). Serial sections were collected and placed on glass slides. The coverslips were then applied to the slides with distrene–plasticiser–xylene (DPX).

#### Hair quantification

**Figure 2:**
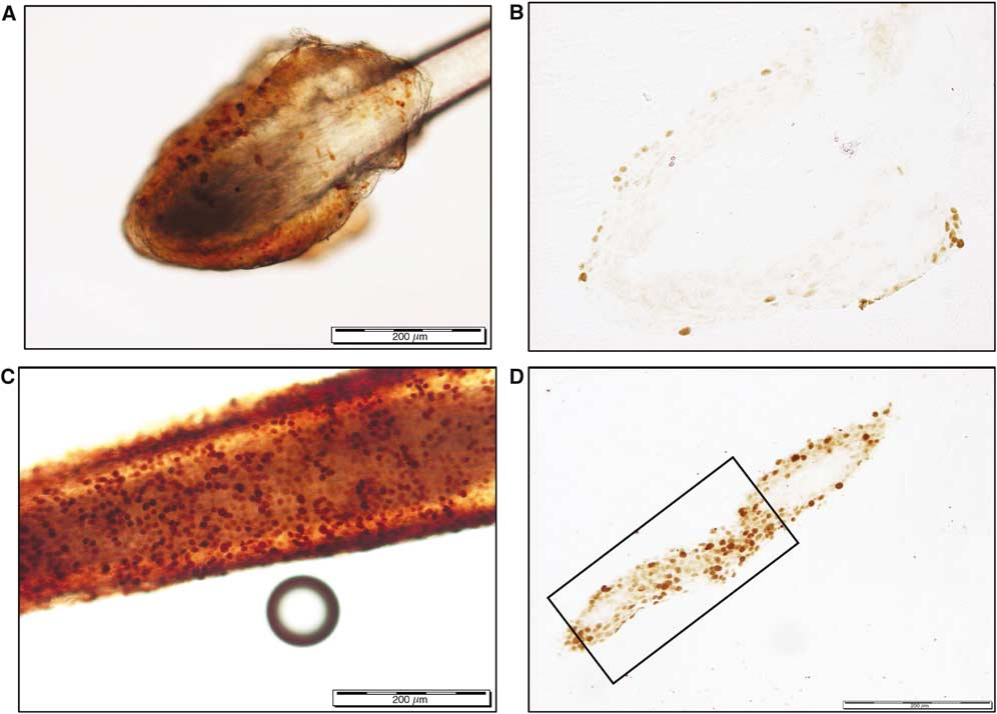
Representative staining of whole eyebrow hair with antibodies directed against Ki67, showing punctate nuclear staining (**A**). Tangential section of same eyebrow hair after embedding in resin used for Ki67 quantification (**B**). Representative staining of whole scalp hair (stage 1) with antibodies directed against Ki67, showing punctate nuclear staining (**C**). Tangential section of same scalp hair after embedding in resin, showing delineated area nearest to bulb on the selected section used for Ki67 quantification (**D**). The scale bars represent 200 *μ*m.

The slides were examined by light microscopy, and three tangential sections from each hair sheath, at the level nearest to the bulb, with clear positive labelled nuclei and minimal background staining were identified and quantified (Figure 2). The total area of the sheath section evaluated and the number of positive labelled nuclei on each of these three sections were assessed using a KS400, version 3.0, image-analysis system within the area of the screen at × 20 magnification. Data from each section were recorded as the number of positive nuclei mm^−2^.

### Statistical analysis

A number of different analysis of variance (ANOVA) models were explored for these data in order to investigate site and stage effects separately. Data were collected on three adjacent sections from each hair, and averaged before analysis.

In all analyses, log-transformed data were used. Zero counts (and missing values due to high background) were substituted with the minimum observed level for each marker *in lieu* of a limit of quantification (LoQ). A statistical significance level of *P*<0.05 was used.

## RESULTS

Even when the length of hair protruding from the scalp was only 5 mm, the length of plucked hair, including the bulb and sheath, was often double this, making handling of the hairs relatively easy. All six markers produced discrete nuclear staining on the resin-embedded sections within both the scalp and eyebrow hairs (cf. Figure 2 for representative Ki67 sections), with no staining present on the negative controls.

Each marker was measured in a total of 40 scalp hairs from eight different subjects and 20 eyebrow hairs from four different subjects. The proportions of hairs providing signals quantified as greater than zero, and the drop-off rates of hairs at each point in the preparation procedure are presented in Table 1.

An ANOVA model with random effect term for subject, and fixed effect term for site, was fitted to the mean quantitative score per hair data to explore the effect of body site (scalp *vs* eyebrow) for each marker. The results of this analysis are shown in Table 2.

The numbers of scalp hairs stained and staged within each classification 0–3, based on the distance from the lower end of the sheath to the base of the bulb, are shown in Table 3. All eyebrow hairs visualised had the sheath around the bulb, and therefore were not individually staged using this system.

An exploratory indication of comparative variation between hairs from the same subjects at the same time, and from between different subjects, was possible using the mean marker levels per hair. The effect on intra- and intersubject coefficients of variation (CV) of differences between stages was investigated by comparing components of variation when the ANOVA model included or excluded stage as a fixed factor (Table 4).

## DISCUSSION

Ki67-positive cells have previously been demonstrated within the outer root sheath of plucked human hairs by immunofluorescence (Gho *et al*, 2004), and epithelial outgrowth has been demonstrated in explant cultures of such plucked hairs *in vitro* (Moll, 1996). We looked at six different cell-cycle-related nuclear markers by IHC in plucked human scalp and eyebrow hair from healthy male volunteers. The proportions of suitable hairs from scalp and eyebrow that were lost or damaged during processing were 18 and 10%, respectively (Table 1). For fully processed scalp and eyebrow hairs across all markers, a total of 20 and 44%, respectively, were also unquantifiable either due to a zero positive nuclear count or due to high background staining (Table 1). It is impossible from this data set to distinguish between unquantifiable counts due to processing failures and genuine biological zeros. However, the relative positive nuclear count frequencies for the different markers in other healthy human tissues are fully consistent with the majority of these zero counts being ‘biological’. Whether low levels of markers could be reliably increased prestudy in order to make them more practicable, for example, through the use hair stimulants such as minoxidil (Messenger and Rundegren, 2004), is currently unknown. The final evaluation of the usefulness of any of these markers as study end points will depend on demonstrating PD activity on them within a future drug-intervention study. We would suggest that in any future drug-intervention study involving PD end points in plucked human hair, zero counts and high background hairs are also fully recorded and analysed in case the effect of the drug is primarily to increase the proportion of biological zeros among the hairs. Zero counts due to processing failures, should, in theory, remain constant over time.

With regard to the body site with the highest marker levels from which to pluck hairs, there is a clear statistically significant difference that favours scalp over eyebrow for Ki67, total p27 and phospho-p27, with all other markers also having higher expression in scalp hairs than in eyebrows (Table 2).

The normal human hair cycle can be divided into three separate phases: anagen, catagen and telogen (Sperling, 1991). During pilot work for the current study, it was apparent that plucked scalp hairs fell within four broadly distinct morphological groups, which we referred to as stages 0–3, based on the distance of the sheath from the base of the bulb under light microscopy (Figure 1). While the stage 0 hairs fitted the description of clubbed telogen hairs very well, stages 1–3 were thought to represent the impact of anatomical weak points within the anagen follicle favouring breakage of the inner and/or outer sheath at particular distances from the base of the bulb. Since there is some evidence that different sections along the sheath of plucked human hair may have different proliferative potentials (Moll, 1996), we developed a system to classify prospectively all hairs within the current study into stages 0–3 (Table 3), looking for any influence of these different stages on the quantitative IHC results.

Given the caveat that there were often small numbers of hairs present within each stage, while there was a difference in some marker levels between hairs at different stages, the pattern was not entirely consistent across all markers, and, in the majority of cases, failed to reach statistical significance (data not shown).

Initial estimates of the intra- and intersubject components of variance for the different markers in scalp hair are shown in Table 4. It is likely that the reliability of these CV estimates could be improved further if a larger data set were used in terms of the number of individuals or number of hairs per individual, or if multiple sections from each hair were counted for each marker, particularly if the sections were stepped to avoid the same cell being counted more than once. It can be seen by comparing corresponding cells in Table 4 that, even when the hair stage effect is highly significant (e.g. total pRb – *P*<0.001 for stage difference), the improvement in intrasubject CV due to the stage adjustment is modest, with a greater impact on intersubject CV, perhaps reflecting a greater variation in the distribution of stages between subjects. Therefore, it seems unlikely that allowing for the effect of stage would make much difference in future studies primarily using within-subject comparisons. The potential impact of factors such as ethnicity and gender, or of any relevant pre-study treatment, for example, whether hair follicles are synchronised in anagen during the recovery from cytotoxic chemotherapy-induced alopecia, is currently unknown.

In conclusion, we have demonstrated the feasibility of detecting and quantifying a range of cell-cycle-related nuclear markers in plucked human hair, which could be used as PD end points in intervention studies using drugs that interact with these pathways.

## Figures and Tables

**Table 1:** Proportion of quantifiable hairs (‘success rate’) by marker from scalp and eyebrow

	**Plucked**	**Stained**	**Through to resin**	**Lost/damaged**	**Zero count**	**High background**	**Quantified >0 (% of total)**
*Scalp*							
Ki67	40	35	35	6	0	0	34 (85%)
Total pRb	40	36	32	5	4	0	31 (78%)
Phospho-pRb	40	37	24	5	14	1	20 (50%)
Total p27	40	35	30	10	1	0	29 (73%)
Phospho-p27	40	36	21	10	12	1	17 (43%)
Phospho-HH3	40	33	27	8	6	10	16 (40%)
							
Total	240	212	169	44 (18%)	37 (15%)	12 (5%)	147 (61%)
							
*Eyebrow*							
Ki67	20	20	18	2	2	0	16 (80%)
Total pRb	20	19	19	1	0	0	19 (95%)
Phospho-pRb	20	20	13	0	7	2	11 (55%)
Total p27	20	20	8	1	11	1	7 (35%)
Phospho-p27	20	20	4	0	20	0	0 (0%)
Phospho-HH3	20	14	10	8	6	4	2 (10%)
							
Total	120	113	72	12 (10%)	46 (38%)	7 (6%)	55 (46%)

Phospho-p27=phosphorylated p27; phospho-pRb=phosphorylated pRb; phospho-HH3=phosphorylated histone H3.

The numbers of hairs in the four columns on the right-hand side add up to the total number of hairs plucked. Hairs could be registered as lost/damaged, or quantified as having no staining, either before or after being taken through to resin.

**Table 2:** Mean per hair data analysis by site (scalp/eyebrow)

	**Scalp glsmean** **(nuclei mm^−2^)**	**Scalp *N* (number** **of hairs)**	**Eyebrow glsmean** **(nuclei mm^−2^)**	**Eyebrow *N* (number of** **hairs)**	***P*-value for site** **difference**
Ki67	4535.1	34	2405.0	18	0.009
Total pRb	6144.1	35	5749.9	19	0.837
Phospho-pRb	2124.3	35	1383.7	20	0.554
Total p27	7903.9	30	2427.8	20	<0.001
Phospho-p27	1783.8	30	929.8	20	0.023
Phospho-HH3	871.0	32	397.3	12	0.198

Phospho-p27=phosphorylated p27; phospho-pRb=phosphorylated pRb; phospho-HH3=phosphorylated histone H3.

The data included zero counts and high background counts expressed as LoQ values.

**Table 3:** Number of hairs within each stage classification for scalp hairs based on distance of the sheath from the base of the hair bulb (*n*=200 hairs stained and staged)

	**Stage 0**	**Stage 1**	**Stage 2**	**Stage 3**
All subjects (*n*=200)	13 (7%)	50 (25%)	79 (40%)	58 (29%)
Per subject ranges	(0–18%)	(5–56%)	(5–80%)	(0–85%)

In parentheses, each stage is also shown as a percentage of the total number of hairs acquired from all subjects, and as the range of percentages seen within individual subjects.

**Table 4:** Variance components of mean per scalp hair data analysis allowing for, or ignoring stages 0–3

	**Intersubject**		**Intrasubject**	
	**CV (%) allowing for** **hair stage**	**CV (%) ignoring** **hair stage**	**CV (%) allowing for** **hair stage**	**CV (%) ignoring** **hair stage**
Ki67	21	32	55	57
Total pRb	23	49	62	75
Phospho-pRb	196	205	42	41
Total p27	0	0	37	41
Phospho-p27	50	48	55	67
Phospho-HH3	113	94	133	135

CV=coefficients of variation; phospho-p27=phosphorylated p27; phospho-pRb=phosphorylated pRb; phospho-HH3=phosphorylated histone H3.

The data included zero counts and high background counts expressed as LoQ values. Of note, with the ANOVA models used, figures for intersubject variance describe the CVs over and above that of the other components of variance, including intrasubject. Consequently, a lower intersubject than intrasubject CV should not be taken as advising against crossover trial designs; for parallel group trial designs would involve elements of both intra- and intersubject components of variance in pretrial sizing decisions. While intersubject CV values should, conceptually, always be greater than zero, as a consequence of the mixed-model analysis technique used, variance components can occasionally be estimated as zero.
